# Supplementary material for: Cryo-EM structure of the NDH–PSI–LHCI supercomplex from Spinacia oleracea
Source: Nat Struct Mol Biol. 2025 Jan 24;32(6):968–78. doi: 10.1038/s41594-024-01478-1 (PMC12170339; doi:10.1038/s41594-024-01478-1)
Supplement: Supplementary file 1 — Supplementary Figs. 1–17 and Table 1. [file 41594_2024_1478_MOESM1_ESM.pdf]

nature structural & molecular biology

Supplementary information https://doi.org/10.1038/s41594-024-01478-1

Cryo-EM structure of the NDH–PSI–LHCI supercomplex from *Spinacia oleracea*

In the format provided by the authors and unedited

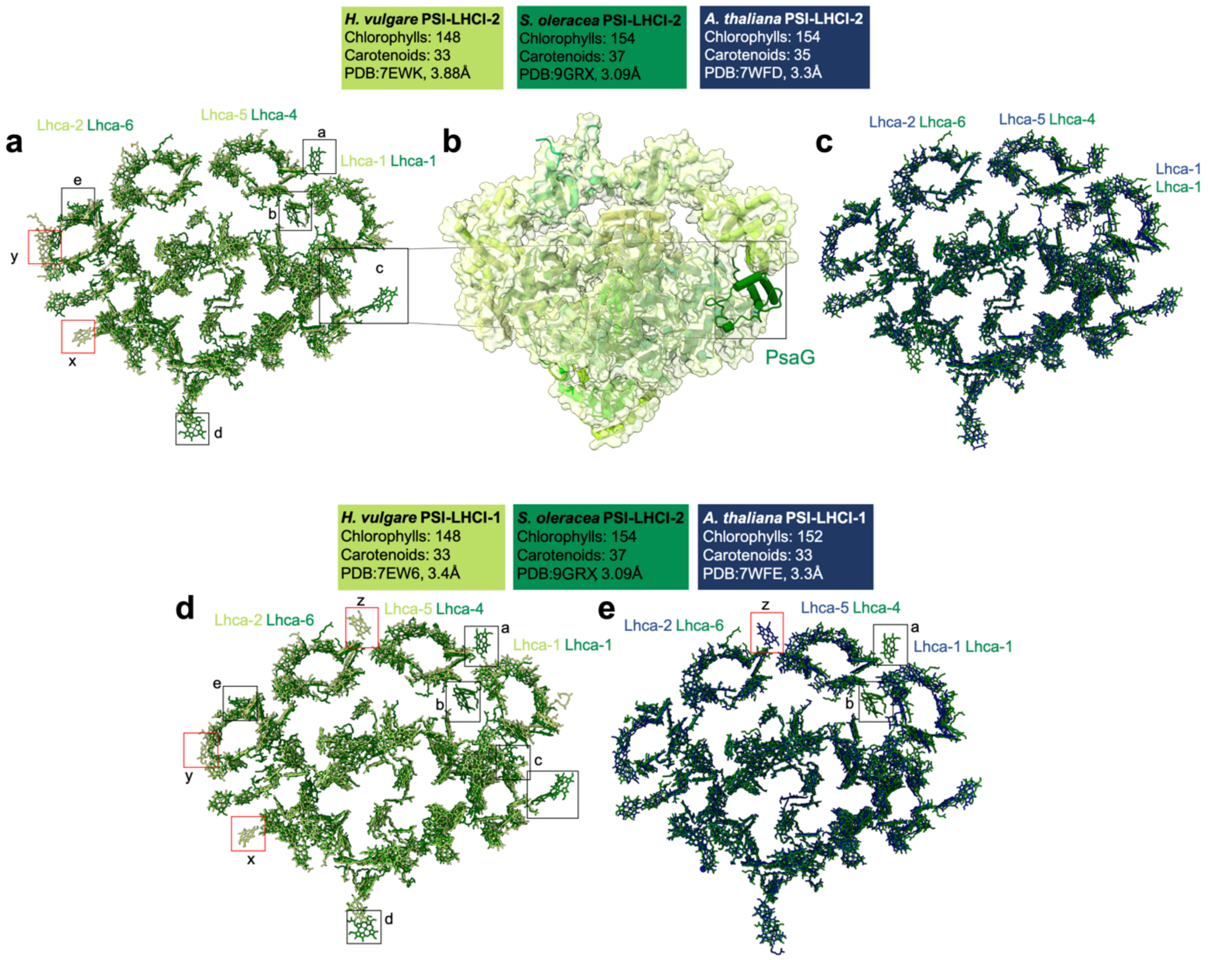

Supplementary Fig. 1: Comparison between PSI-LHCIs from spinach, barley and *A. thaliana* suggests that chlorophylls bind and reorganize according to subunits composition and species of origin. We refer to the complexes as PSI-LHCI-1 or PSI-LHCI-2 as specified in Extended Data Fig. 2. Chlorophylls in PSI-LHCI-2 from *S. oleracea* compared to PSI-LHCI-2 (**a**) from barley and (**c**) *A. thaliana*, and to PSI-LHCI-1 (**d**) from barley and (**e**) from *A. thaliana.* (**c**) The superposition of PSI-LHCI-2 from spinach (cartoon, helices shown as cylinders) with PSI-LHCI-2 from barley (green-yellow surface) reveals that subunit PsaG is missing in barley (this subunit is absent also in PSI-LHCI-1from barley). (**a, d, e**) Red squares highlight extra chlorophylls that are found in barley and *A. thaliana* but not in spinach; black squares indicate the position of chlorophylls observed only in spinach. Another persistent difference between our PSI-LHCI-2 and both PSI-LHCI-1 is an extra chlorophyll between Lhca-2 and Lhca-5 (red square z in supplementary figures **d** and **e**). The absent or extra chlorophylls in PSI-LHCI-1 are close to Lhca-5 (the equivalent of Lhca-6) which is involved in the interaction with NDH. Thus, we assume that their presence or absence in this position may be related to energy transfer in PSI-LHCI-1.

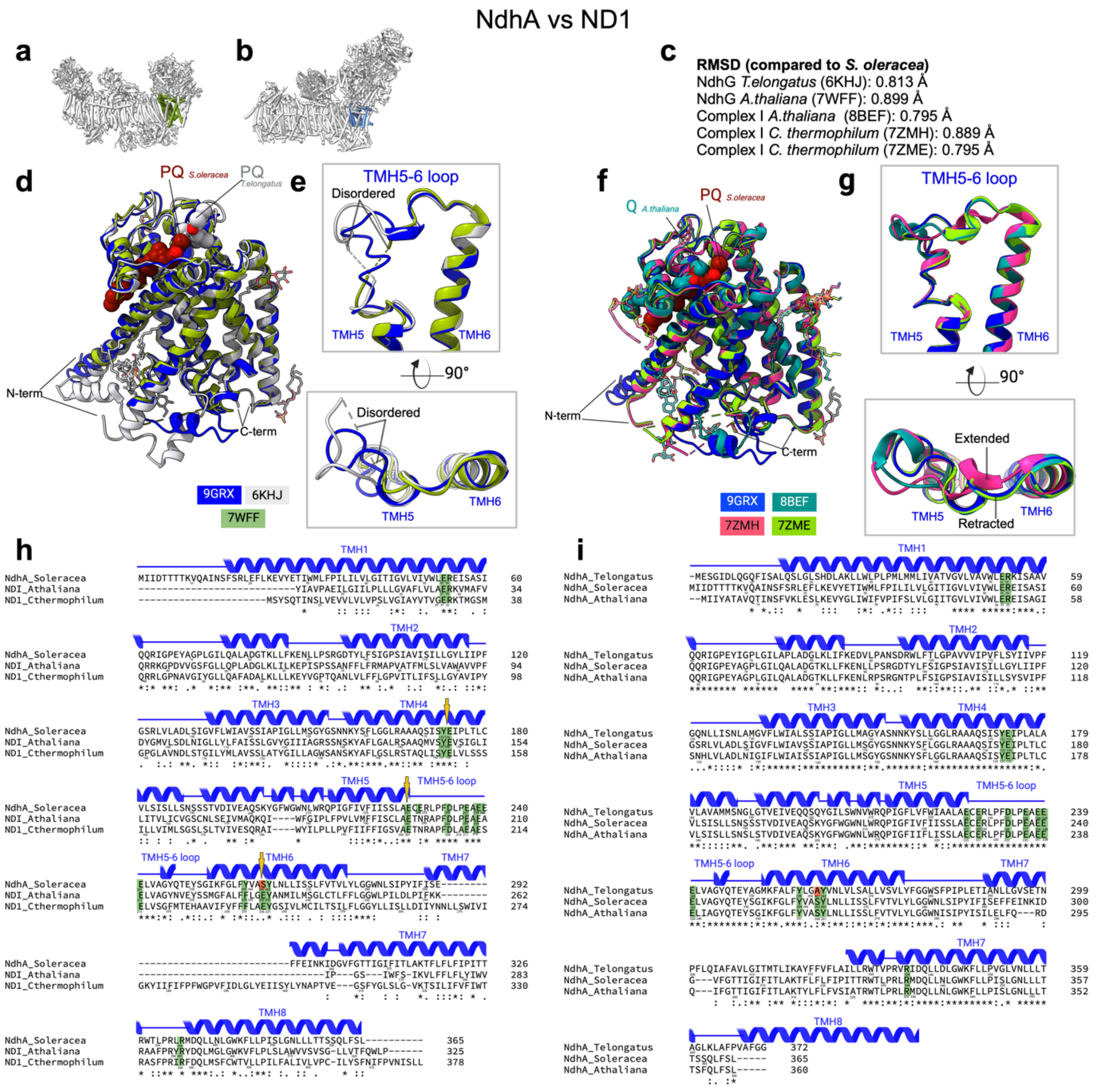

Supplementary Fig. 2: Comparison of NDH core subunit NdhA from *S. oleracea* with NdhA from different organisms and the homologous ND1 from complex I. a, and **b**, Models of NDH (**a**) and Complex I (**b**, PDB ID: 7AR7) with highlighted NdhA and ND1. **c,** Values of CαRMSD (Å) between the indicated chains. **d-g**, Superimposition of the model of the NDH NdhA subunit from spinach (blue) with **(d)** NDH NdhA subunits from *A. thaliana* (green, PDB ID: 7WFF) and *T. elongatus* (light grey, PDB ID: 6KHJ), and (**f**) complex I ND1 subunits from *A. thaliana* (teal, PDB ID: 8BEF) and the two states from *C. thermophilus* (strawberry and lime PDB IDs: 7ZMH and 7ZME). **e,** and **f**, Highlight on the TMH5-6 loop from lateral (top panel) and top views (bottom panel), as described by Laube et al., 2022. **h,** and **i**, Comparison of the *S. oleracea* NdhA protein sequence with the

homologous subunit from specified organisms. Secondary structures representations align with those of Spinach NdhA. Alignment conducted using ClustalOmega[70]. An asterisk indicates positions of single, fully conserved residues. A colon indicates conservation between strongly similar groups. A dot indicates weakly conserved groups. Residues known to play key roles in proton translocation in complex I are highlighted in a color-coded manner. When these residues are conserved across the proteins, they are highlighted in green. Non-conserved residues are highlighted in red. The yellow arrows highlight residues of glutamate on TMH6 that in complex I are involved in proton translocation[42] (**h**). This residue is substituted by a serine in spinach and *A. thaliana* (**i**).

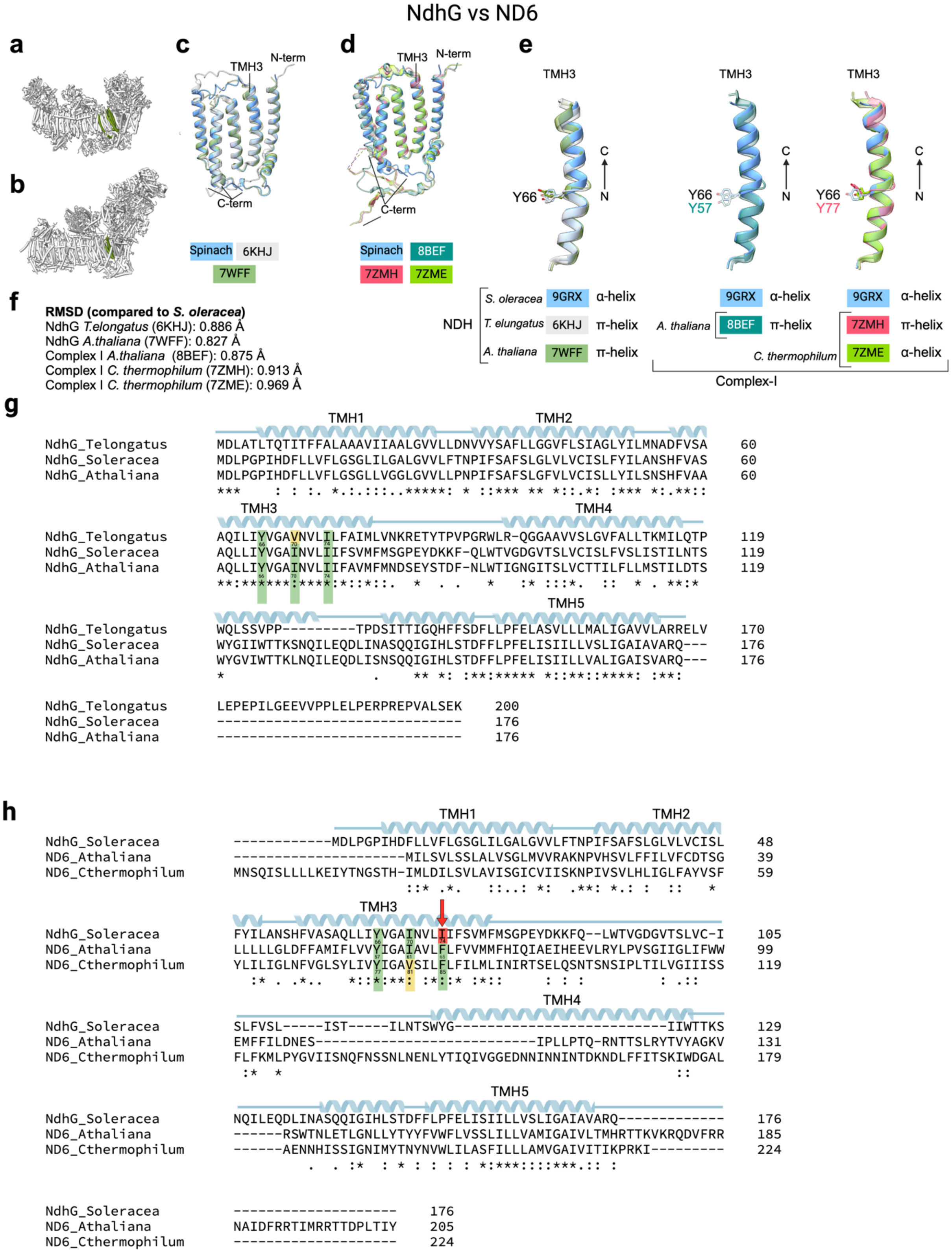

Supplementary Fig. 3: Comparison of NDH core subunit NdhG from S. oleracea with NdhG from different organisms and the homologous ND6 from complex I. a, and **b**, Models of NDH **a,** and Complex I (**b**, PDB ID: 7AR7) with highlighted NdhG and ND6. **c,**

and **d**, Superimposition of the model of the NDH NdhG subunit from spinach (light blue) with **d,** NDH NdhG subunits from *A. thaliana* (green, PDB ID: 7WFF) and *T. elongatus* (light grey, PDB ID: 6KHJ), and **f,** complex I ND6 subunits from *A. thaliana* (teal, PDB ID: 8BEF) and the two states from *C. thermophilus* (strawberry and lime PDB IDs: 7ZMH and 7ZME). **e,** Highlight on TMH3 that constitutes the so called π-gate. Conserved key residues are shown as sticks. **f,** Values of CαRMSD (Å) between the indicated chains. **g,** and **h**, Comparison of the *S. oleracea* NdhG protein sequence with the homologous subunit from specified organisms. Secondary structures representations align with those of Spinach NdhG. Sequences were aligned with ClustalOmega[70]. An asterisk indicates positions of single, fully conserved residues. A colon indicates conservation between strongly similar groups. A dot indicates weakly conserved groups. Residues known to play key roles in proton translocation in complex I are highlighted. Conserved residues among the species are highlighted in green while non-conserved in red. Residues in yellow indicate a conservative replacement.

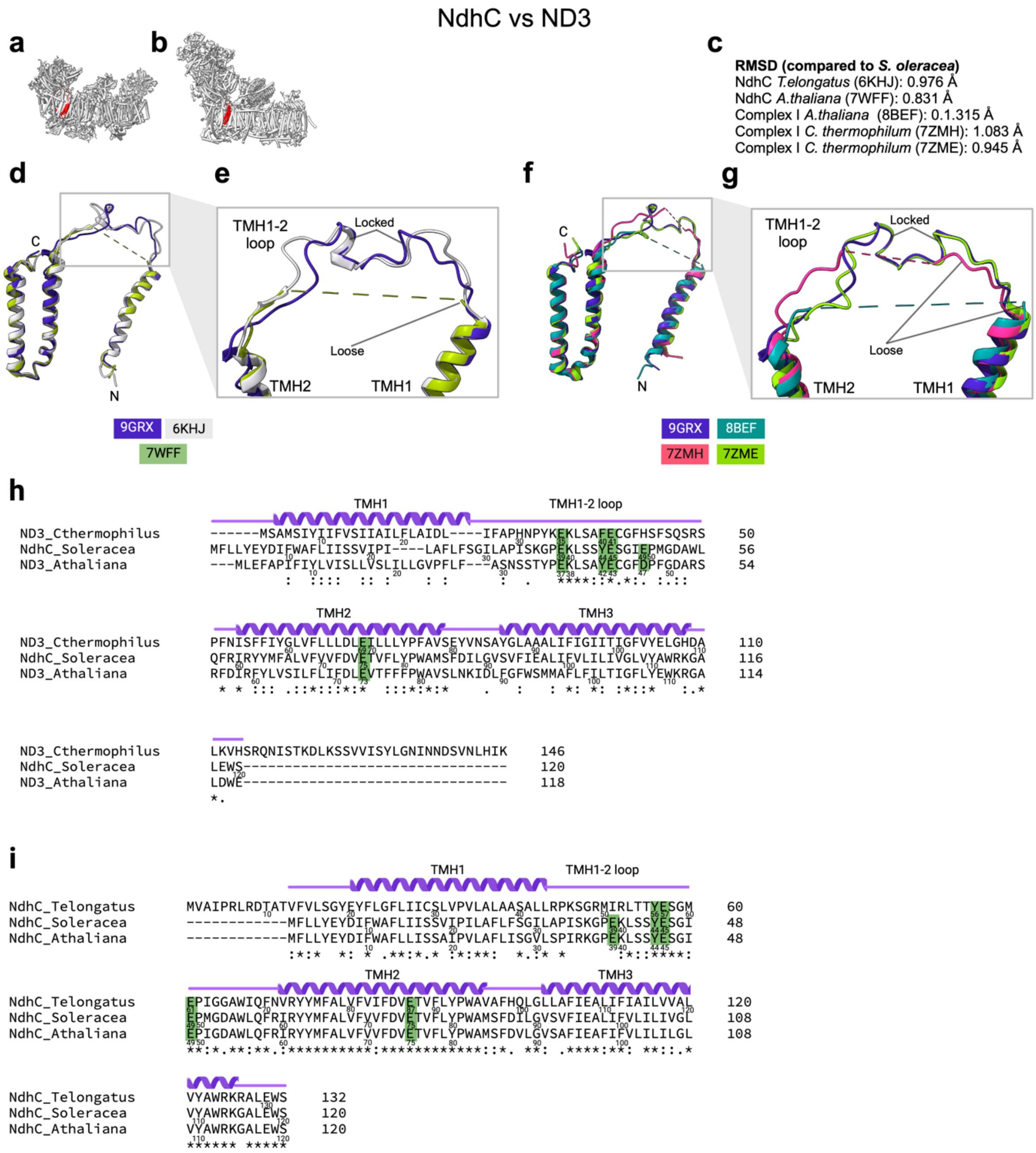

Supplementary Fig. 4: Comparison of NDH core subunit NdhC from S. oleracea with NdhC from different organisms and the homologous ND3 from complex I. a, and **b**, Models of NDH **a,** and Complex I (**b**, PDB ID: 7AR7) with highlighted NdhC and ND3. **c,** Values of CαRMSD (Å) between the indicated chains. **d-g**, Superimposition of the model of the NDH NdhC subunit from spinach (purple) with **d,** NDH NdhC subunits from *A. thaliana* (green, PDB ID: 7WFF) and *T. elongatus* (light grey, PDB ID: 6KHJ), and (**f**) complex I ND3 subunits from *A. thaliana* (teal, PDB ID: 8BEF) and the two states from *C. thermophilus* (strawberry and lime PDB IDs: 7ZMH and 7ZME). **e** and **f**, Highlight on the

TMH1-2 loop from a lateral view. Loop arrangement is described according to Laube et al., 2022. **h,** and **i**, Comparison of the *S. oleracea* NdhC protein sequence with the homologous subunit from specified organisms. Secondary structures representations align with those of Spinach NdhC. Sequences were aligned with ClustalOmega[70]. An asterisk indicates positions of single, fully conserved residues. A colon indicates conservation between strongly similar groups. A dot indicates weakly conserved groups. Residues known to play key roles in proton translocation in complex I are highlighted: residues conserved across species are highlighted in green.

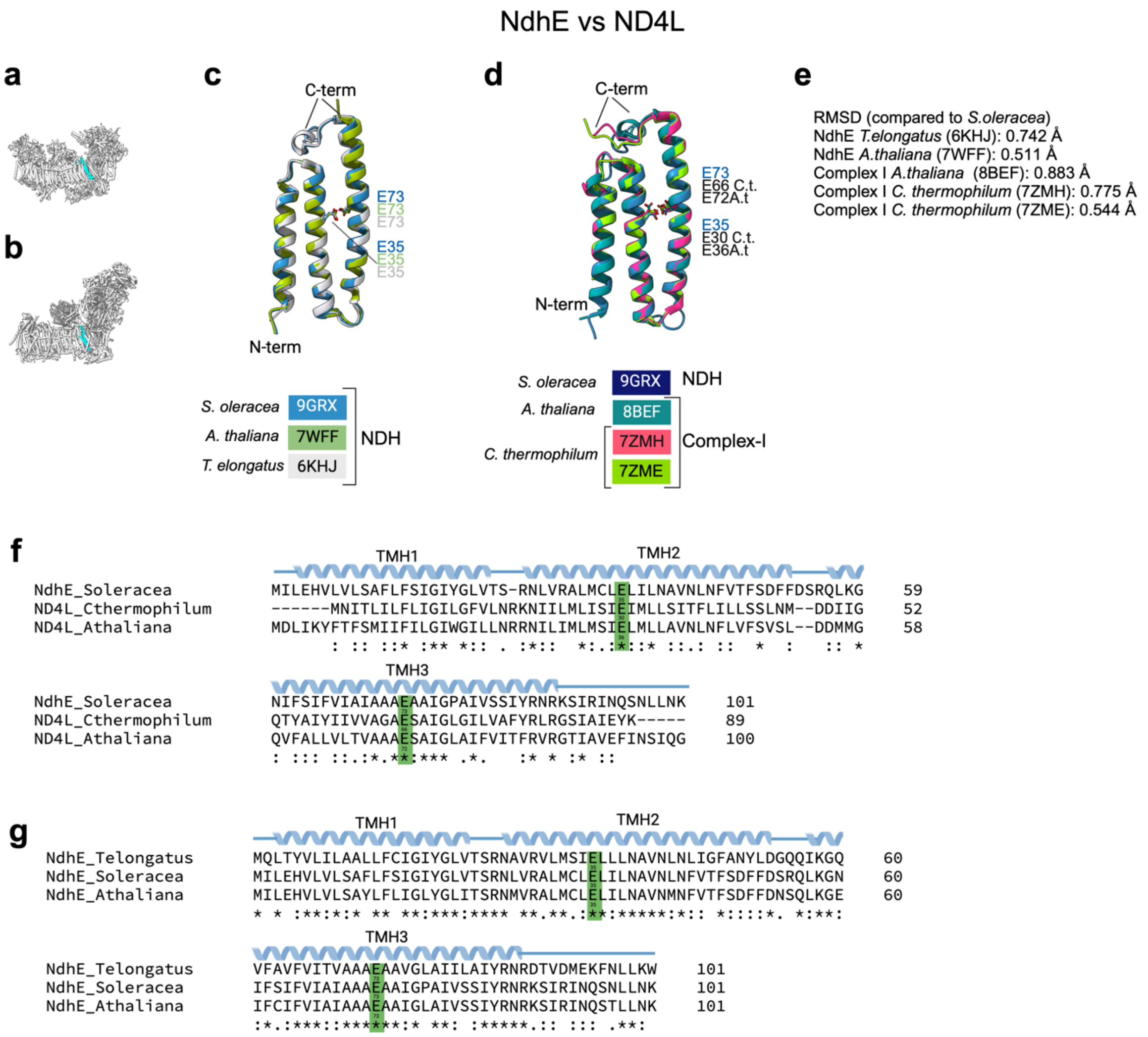

Supplementary Fig. 5: Comparison of NDH core subunit NdhE from S. oleracea with NdhE from different organisms and the homologous ND4L from complex I. a, and **b**, Models of NDH **a,** and Complex I (**b**, PDB ID: 7AR7) with highlighted NdhE and ND4L. **c,** and **d**, Superimposition of the model of the NDH NdhE subunit from spinach (turquoise) with **d,** NDH NdhE subunits from *A. thaliana* (green, PDB ID: 7WFF) and *T. elongatus* (light grey, PDB ID: 6KHJ), and (**f**) complex I ND4L subunits from *A. thaliana* (teal, PDB ID: 8BEF) and the two states from *C. thermophilus* (strawberry and lime PDB IDs: 7ZMH and 7ZME). Conserved key residues are shown as sticks. **e,** Values of CαRMSD (Å) between the indicated chains. **f,** and **g**, Comparison of the *S. oleracea* NdhE protein sequence with the homologous subunit from specified organisms. Secondary structures representations align with those of Spinach NdhE. Sequences were aligned with ClustalOmega[70]. An asterisk indicates positions of single, fully conserved residues. A colon indicates conservation between strongly similar groups. A dot indicates weakly conserved groups. Residues known

to play key roles in proton translocation in complex I and that are conserved among the indicated species are highlighted in green.

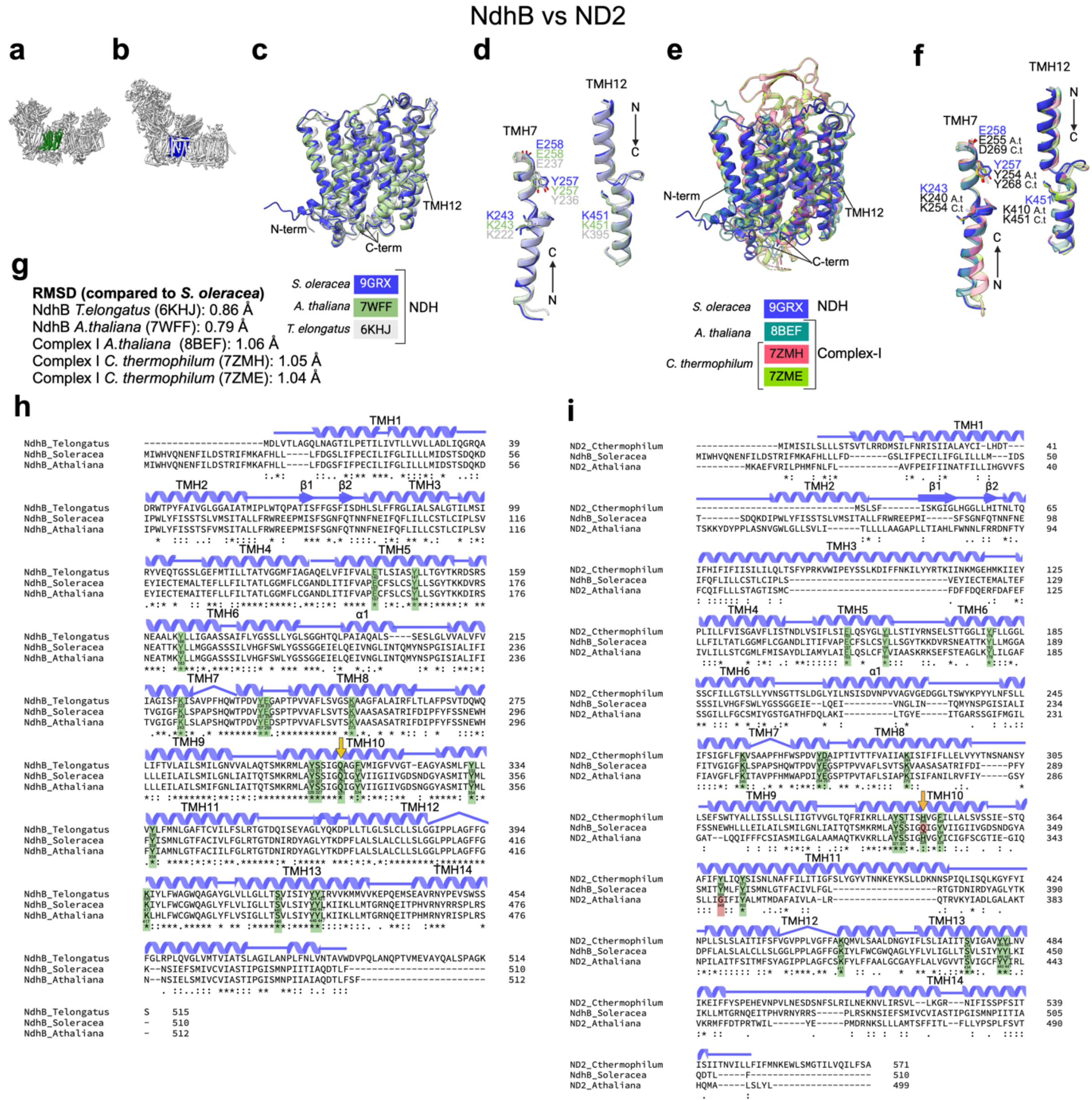

Supplementary Fig. 6: Comparison of NDH core subunit NdhB from S. oleracea with NdhB from different organisms and the homologous ND2 from complex I. a, and **b**, Models of NDH (**a**)**,** and Complex I (**b**, PDB ID: 7AR7) with highlighted NdhB and ND2. **c-f**, Superimposition of the model of the NDH NdhB subunit from spinach (royal blue) with (**d**) NDH NdhB subunits from *A. thaliana* (green, PDB ID: 7WFF) and *T. elongatus* (light grey, PDB ID: 6KHJ), and (**f**) complex I ND2 subunits from *A. thaliana* (teal, PDB ID: 8BEF) and the two states from *C. thermophilus* (strawberry and lime PDB IDs: 7ZMH and 7ZME). **d** and **f**, Highlight on TMH7 and THM12 with conserved key residues shown as sticks. **g,** Values of CαRMSD (Å) between the indicated chains. **h,** and **i**, Comparison of the *S. oleracea* NdhB protein sequence with the homologous subunit from specified organisms. Secondary structures representations align with those of Spinach NdhB. Sequences were

aligned with ClustalOmega[70]. An asterisk indicates positions of single, fully conserved residues. A colon indicates conservation between strongly similar groups. A dot indicates weakly conserved groups. Residues known to play key roles in proton translocation in complex I are highlighted. Conserved residues across the species are highlighted in green while non-conserved in red. Residues in yellow indicate a conservative replacement. The yellow arrow highlights a histidine that is in a key position for proton translocation in complex I (**i**). In NDH it is replaced by a conserved residue of glutamine (**h**).

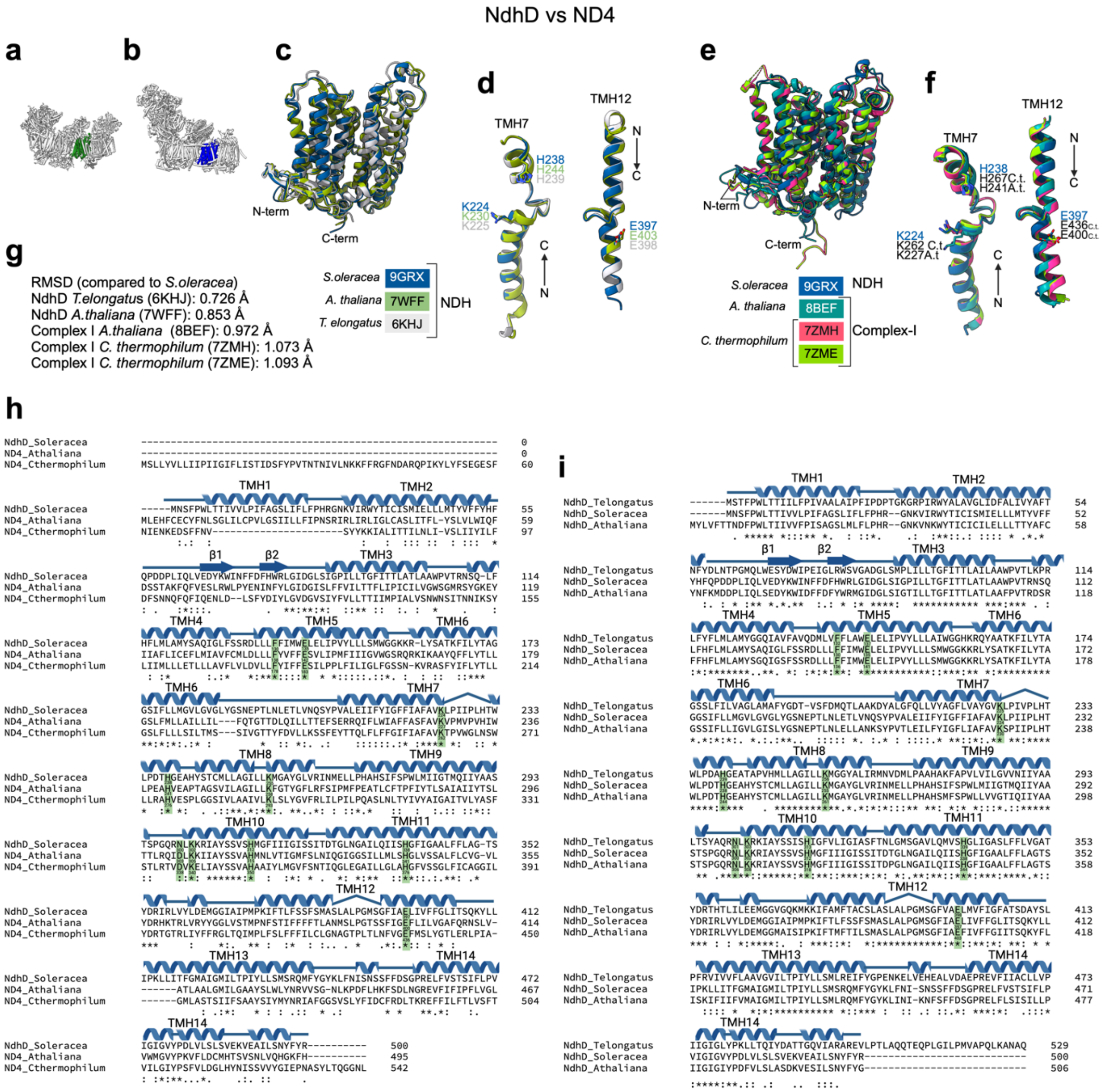

Supplementary Fig. 7: Comparison of NDH core subunit NdhD from S. oleracea with NdhD from different organisms and the homologous ND4 from complex I. a, and **b**, Models of NDH (**a**) and Complex I (**b**, PDB ID: 7AR7) with highlighted NdhD and ND4. **c,** and **e**, Superimposition of the model of the NDH NdhD subunit from spinach (petrol) with **d,** NDH NdhD subunits from *A. thaliana* (green, PDB ID: 7WFF) and *T. elongatus* (light grey, PDB ID: 6KHJ), and (**f**) complex I ND4 subunits from *A. thaliana* (teal, PDB ID: 8BEF) and the two states from *C. thermophilus* (strawberry and lime PDB IDs: 7ZMH and 7ZME). **d,** and **f**, Highlight on TMH7 and THM12 with conserved key residues shown as sticks. **g,** Values of CαRMSD (Å) between the indicated chains. **h,** and **i**, Comparison of the *S. oleracea* NdhD protein sequence with the homologous subunit from specified organisms. Secondary structures representations align with those of Spinach NdhD. Sequences were

aligned with ClustalOmega[70]. An asterisk indicates positions of single, fully conserved residues. A colon indicates conservation between strongly similar groups. A dot indicates weakly conserved groups. Residues known to play key roles in proton translocation in complex I and that are conserved across the species are highlighted in green.

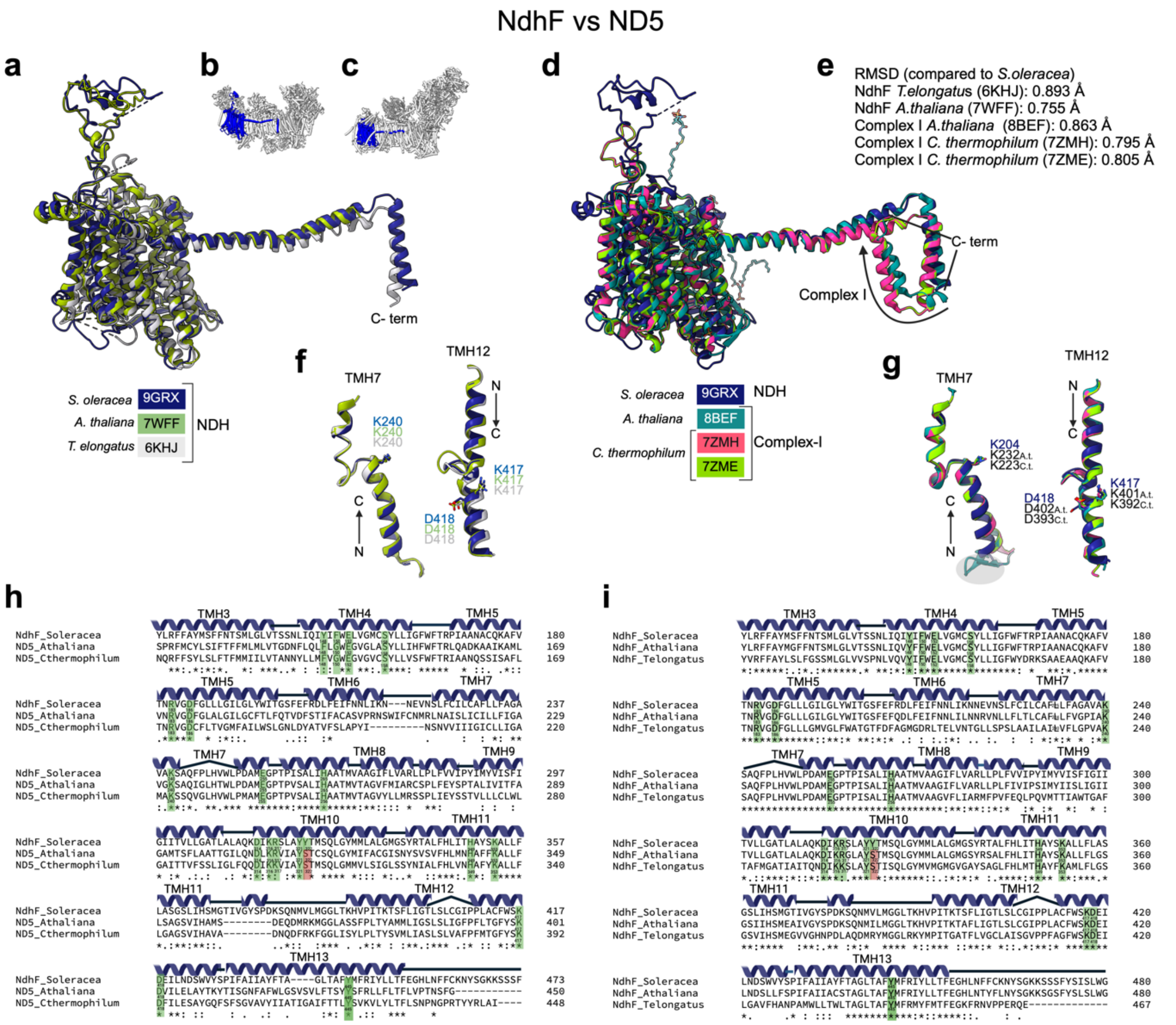

Supplementary Fig. 8: Comparison of NDH core subunit NdhF from S. oleracea with NdhF from different organisms and the homologous ND5 from complex I. b, and **c,** Models of NDH **a,** and Complex I (**b**, PDB ID: 7AR7) with highlighted NdhF and ND5. **a,** and **d,** Superimposition of the model of the NDH NdhF subunit from spinach (dark blue) with **d,** NDH NdhF subunits from *A. thaliana* (green, PDB ID: 7WFF) and *T. elongatus* (light grey, PDB ID: 6KHJ), and (**f**) complex I ND5 subunits from *A. thaliana* (teal, PDB ID: 8BEF) and the two states from *C. thermophilus* (strawberry and lime PDB IDs: 7ZMH and 7ZME). Interestingly, differently from what has been observed in NDH, in complex I the C-terminal part of ND5 forms an extra helix that inserts into the transmembrane region. **f,** and **g,** Highlight on TMH7 and THM12 with conserved key residues shown as sticks. **e,** Values of CαRMSD (Å) between the indicated chains. **h,** and **i,** Comparison of the *S. oleracea* NdhF protein sequence with the homologous subunit from specified organisms. Secondary structures representations align with those of Spinach NdhF. Sequences were aligned with ClustalOmega[70]. An asterisk indicates positions of single, fully conserved residues. A colon

indicates conservation between strongly similar groups. A dot indicates weakly conserved groups. Residues known to play key roles in proton translocation in complex I are highlighted. Conserved residues among the species are highlighted in green while non-conserved in red. Residues in yellow indicate a conservative replacement.

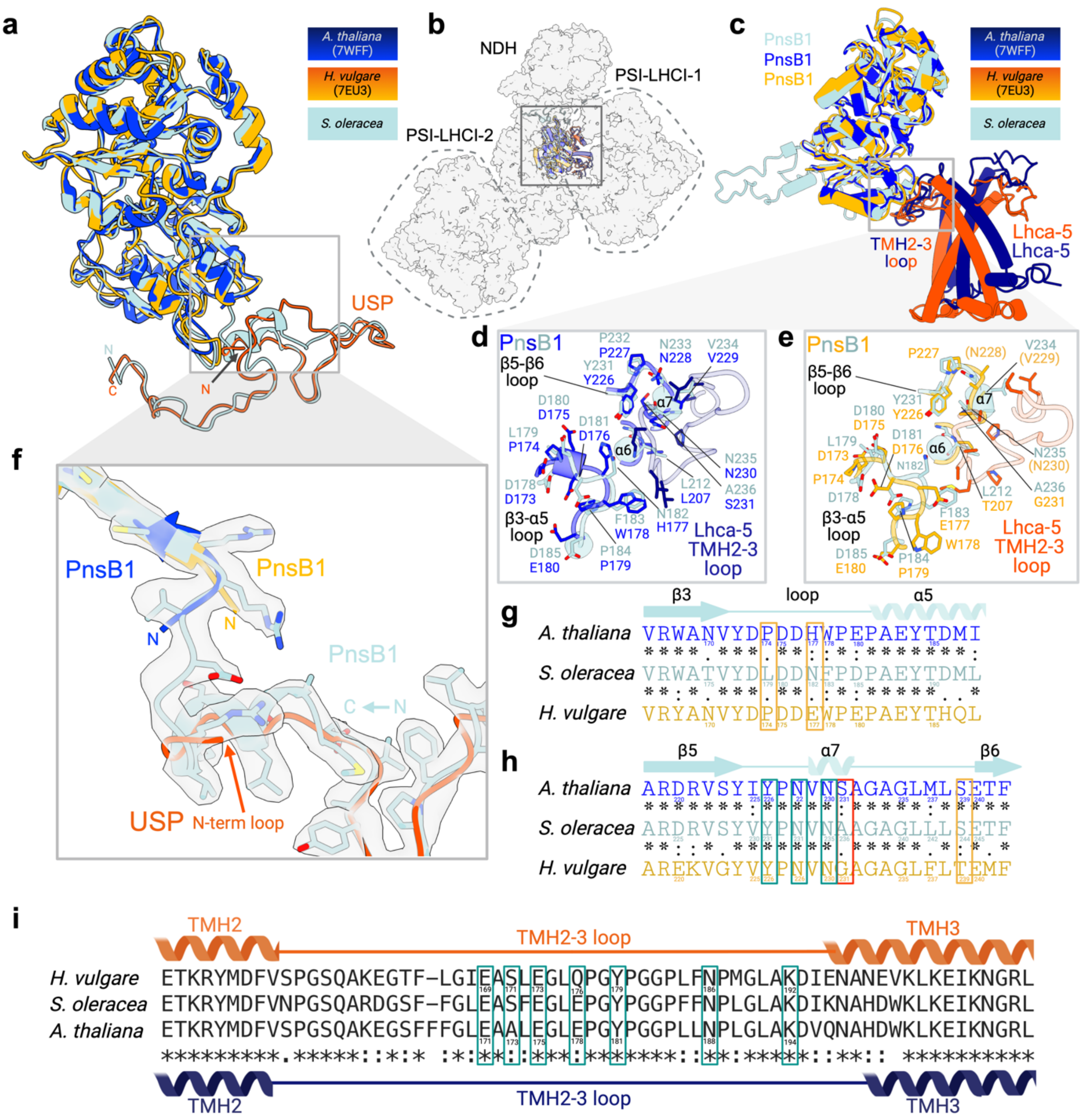

Supplementary Fig. 9: PnsB1 could mediate the interaction with the second PSI in *S. oleracea*. a, Superimposition of subunit PnsB1 from *A. thaliana* and *H. vulga*re to PnsB1 of *S. oleracea*. The structures are highly similar (RMSD compared to spinach: 0.86 for barley and 0.9 for Arabidopsis). **b,** Top view of the cryoEM map from *A. thaliana* (EMD-32477). Models of PnsB1 and Lhca-5 from each species are fitted relatively to *S. oleracea* PnsB1. **c,** PnsB1 and Lhca-5 models showing the main interaction point. Although we did not detect Lhca-5 or a second copy of PSI, we compared our PnsB1 model as indicated to understand if the absence of a second copy of PSI is caused by differences in protein sequence. **d,** and **e**, Superimposition of spinach PnsB1 with secondary structure elements involved in the contacts between PnsB1 and Lhca-5 from (**d**) *A.thaliana* and (**e**) *H. vulgare*. **f,** The long N-terminus of

PnsB1 is the USP protein. The contour level of the *S. oleracea* cryoEM density map is 0.25 (EMD-19246). **g,** and **h**, Comparison of PnsB1 protein sequences of the elements indicated. Secondary structures according spinach PnsB1. **i,** Comparison of TMH2-3 loop sequences from the organisms indicated. The reported Lhca-5 secondary structures are those of PDB ID:7EW6 for *H. vulgare* (orange) and of PDB ID:7WFE for *A. thaliana* (dark blue). **g-i**: sequences aligned with ClustalOmega[70]. An asterisk indicates positions of single, fully conserved residues. A colon indicates conservation between strongly similar groups. A dot indicates weakly conserved groups. Residues that may play a role in the interaction between NDH and the second PSI are highlighted and color-coded. When these residues are conserved across all three species, they are highlighted in green. Those that are not conserved are marked in red. Residues that lack conservation across species but are not positioned in potentially significant locations are mustard-yellow.

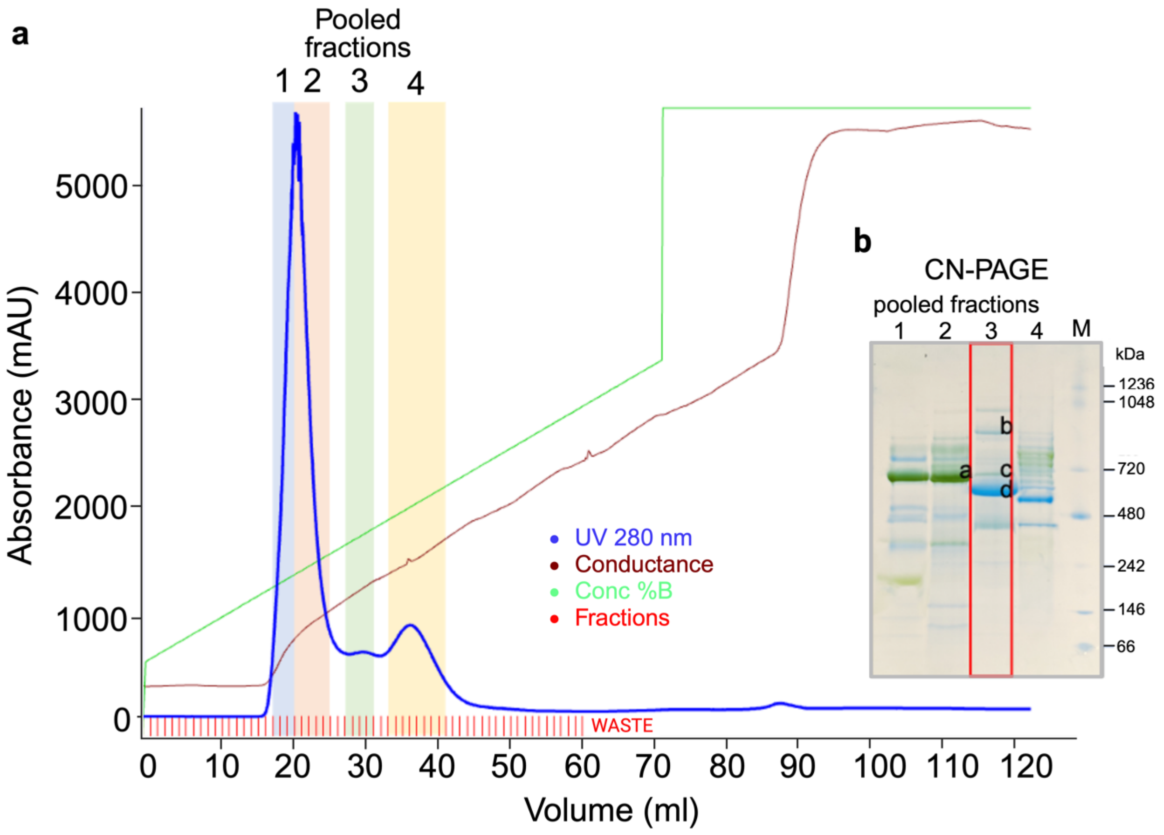

Supplementary Fig. 10: Purification of NDH-PSI-LHCI-2 supercomplex from spinach leaves. **a**, Protein complexes of enriched stroma lamella were solubilized with Buffer containing 1 % (w/v) trans-4-(trans-4'-propylcyclohexyl)cyclohexyl-α-D-maltoside (t-PCCαM) and separated by ion-exchange chromatography. Fractions of main elution peaks were pooled (1 – 4) and **b**, 20 µg total protein od each pool was separated on a CN-PAGE and stained using Coomassie R250 (Blue Silver) to analyze its content. Fractions containing protein complexes were assigned by their migration properties and indicated by color: a, PSII* core; b, NDH-PSI-LHCI-2; c, PS1; d, CF_1F_O. Fraction pool 3 was used for further NDH-PSI-LHCI-2 purification. The purification procedure and the CN-PAGE shown here were performed for both the datasets used in this work. The raw image of the CN-PAGE shown in **b** has not been retained. However, a close-up of the gel without any text overlay is provided in Supplementary Fig. 17**a**.

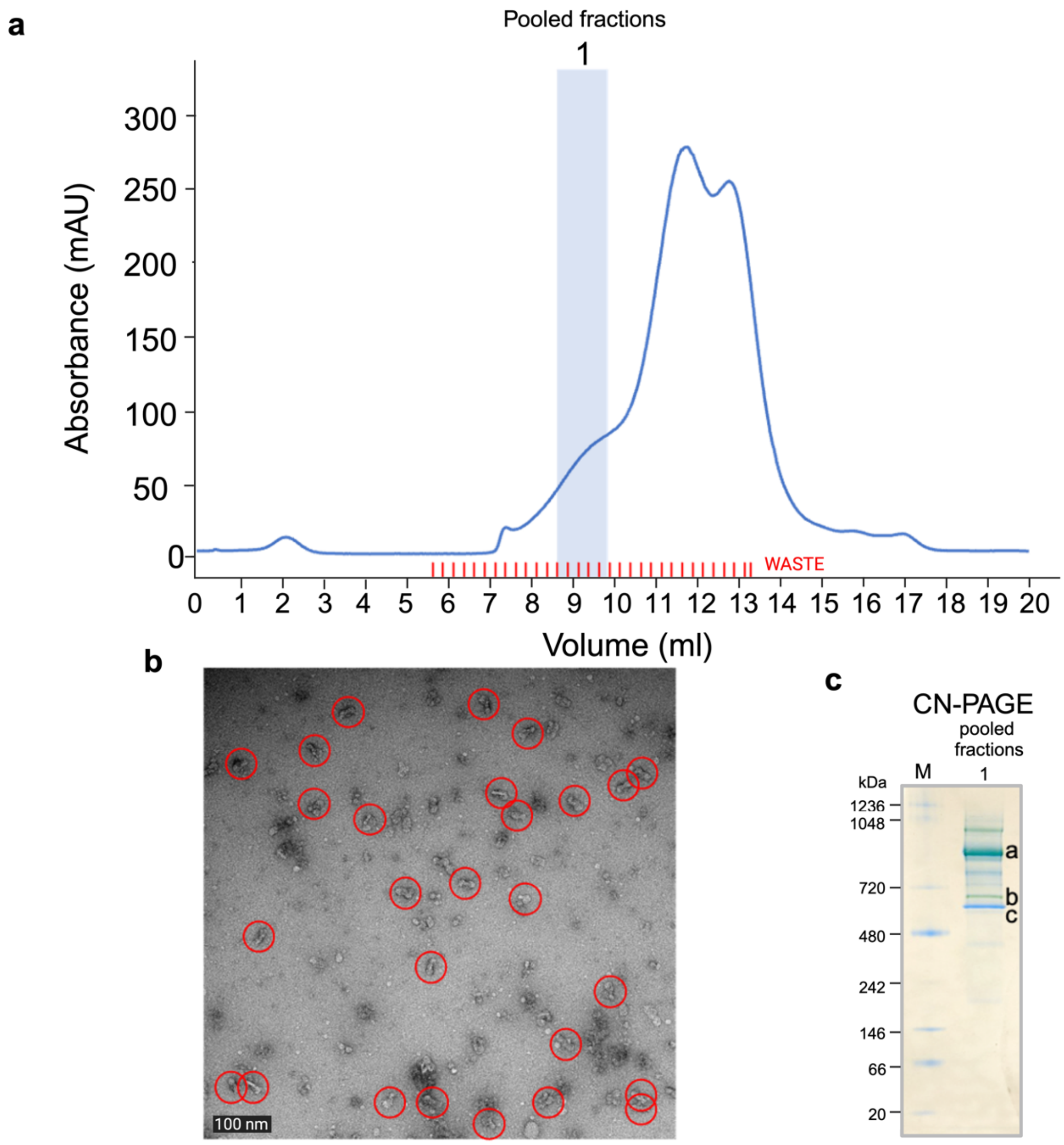

Supplementary Fig. 11: Purification of NDH-PSI-LHCI-2. a, Protein complexes of Pool Fraction 3 were further separated by Superose-6 (GE Healthcare Life Sciences, US) gel-filtration. The content of each peak was analyzed by negative stain electron microscopy to identify protein complexes. The main peaks eluting at 11 and 13 ml contain mostly PS1 and CF_1F_O. **b**, Representative negative stain electron micrograph of NDH-PSI-LHCI-2 of the supercomplex in the first elution peak at ~9 ml. Fractions of this peak were pooled, concentrated and loaded on a CN-PAGE **(c)** for confirmation. **c,** After Coomassie staining, visible bands were assigned by migration behavior and color to: a, NDH-PSI-LHCI-2; b, PS1; c, CF_1F_O. The concentrated sample was used directly for cryoEM sample preparation. The purification procedure and the investigation of the final sample by negative staining and the CN-PAGE shown here were performed for both the datasets used in this work. The raw

image of the CN-PAGE shown in **c** has not been retained. However, a close-up of the gel without any text overlay is provided in Supplementary Fig. 17**b**.

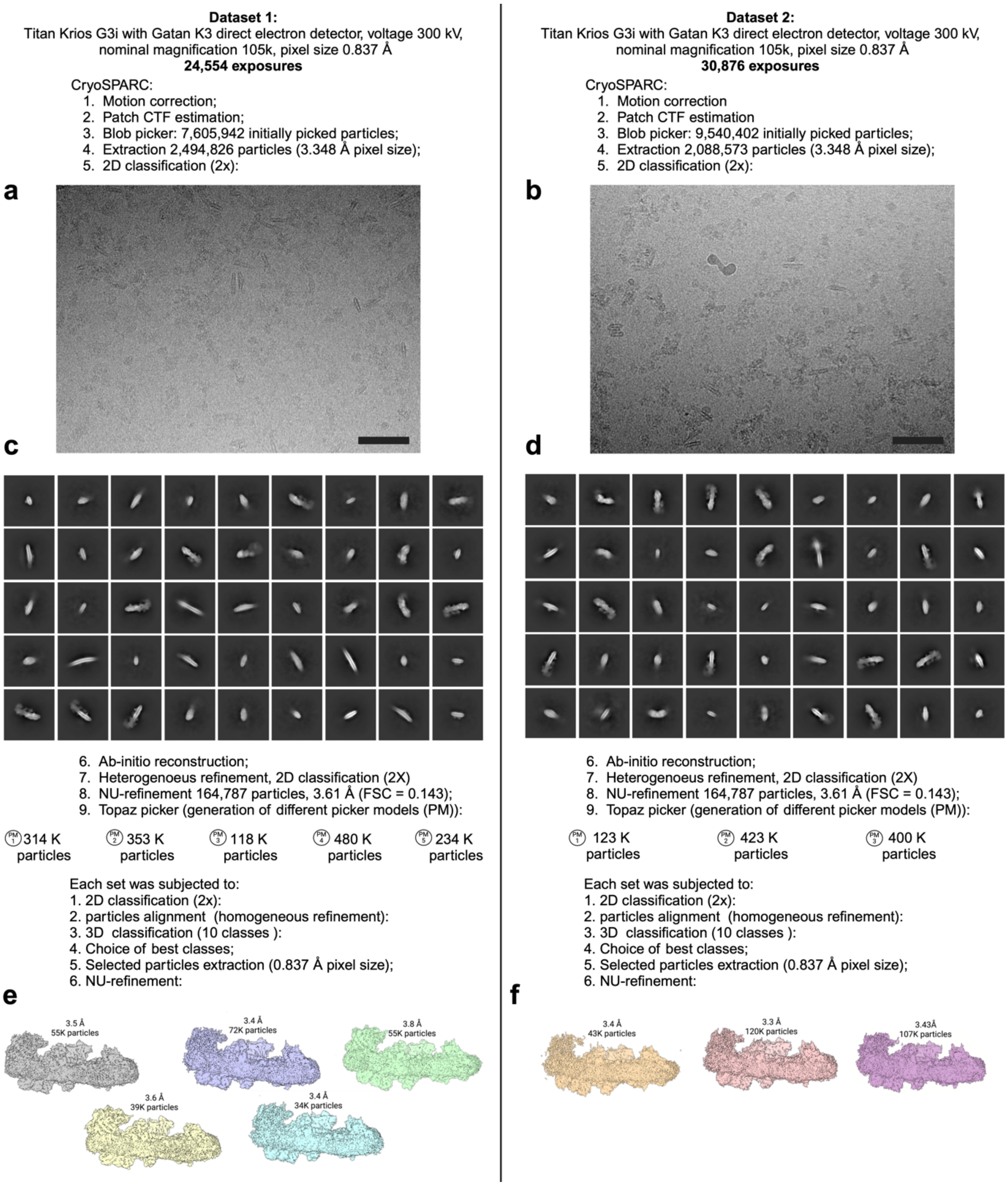

Supplementary Fig. 12: CryoEM processing pipeline for NDH-PSI-LHCI-2. Initial workflow for single-particle image processing applied to the two datasets acquired for this study. **a,** and **b**, Representative micrographs. Scale bar: 100 nm. **c,** and **d**, 2D class averages from the two datasets. **e,** and **f**, CryoEM density maps generated from sets of particles picked using different settings in Topaz.

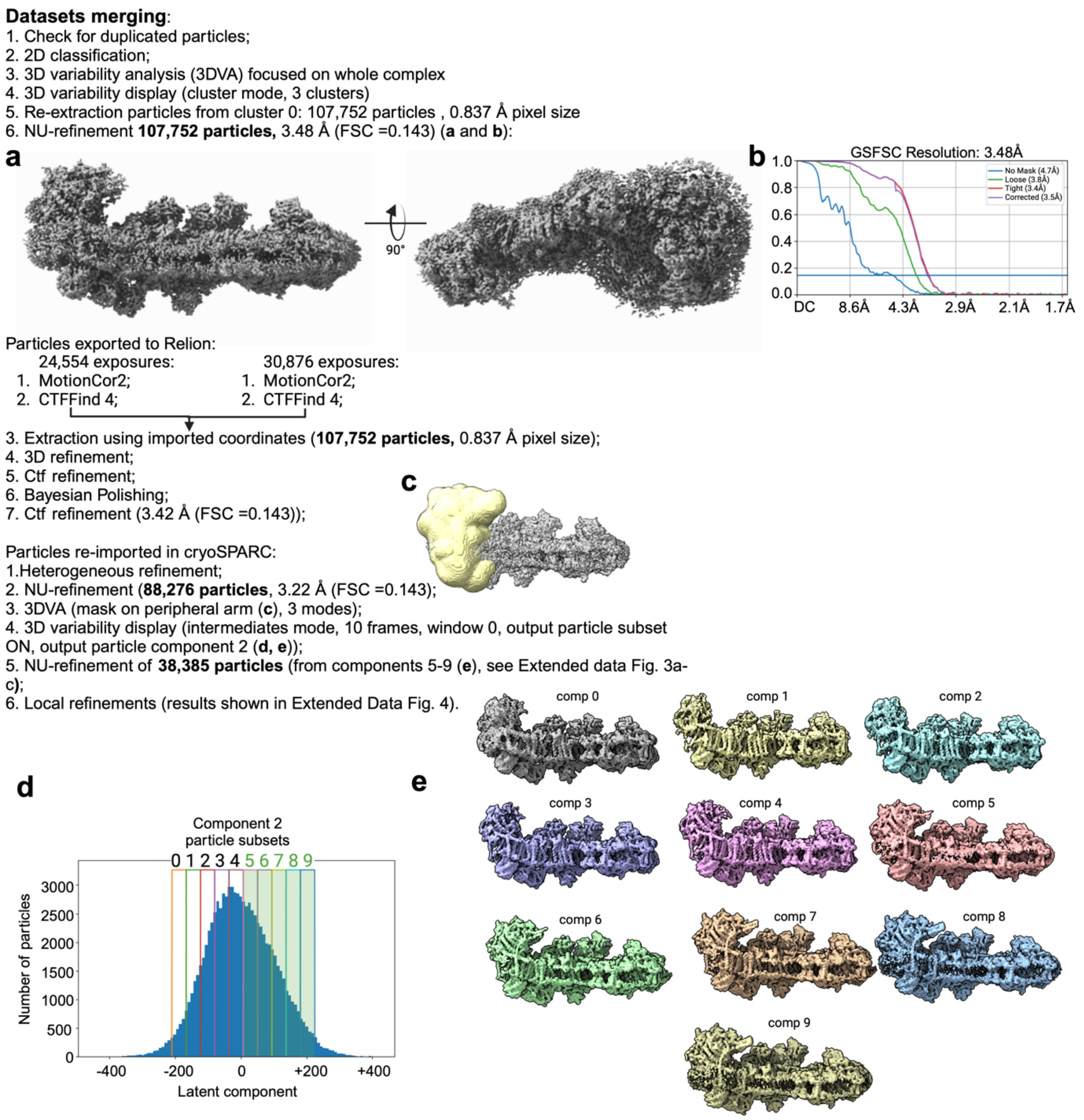

Supplementary Fig. 13: CryoEM processing pipeline and final map for NDH-PSI-LHCI-2. Selected particles from the two datasets, picked using different settings in Topaz (see Supplementary Fig. 9**e**, **f**), were merged. The workflow leading to the final map is detailed. **a,** and **b,** Representative cryoEM map and corresponding FSC curve of NDH-PSI-LHCI-2 before particles selection via 3D variability analysis (3DVA), showing a poorly resolved peripheral arm. **c**, The mask used for 3DVA around the peripheral arm of NDH, is shown in yellow. **d** and **e**, Particle subsets were generated using 3D variability display on component 2 (a total of three modes was solved in 3DVA), utilizing the "intermediates" mode with the "rolling window" set to 0. This setting ensured an even distribution of particles along the chosen component **(d).** Volumes generated from each subset were analyzed in

ChimeraX. Only particles with a well-defined peripheral arm were chosen for further 3D refinement. Maps are shown at a threshold level of 1. From the resulting subsets, subsets five to nine (**d**, highlighted in green and **e**) were selected for further refinement. The 3D reconstruction of the merged particle clusters five to nine resulted in a map with a well resolved peripheral arm of NDH (see Extended Data Fig. 3a-c).

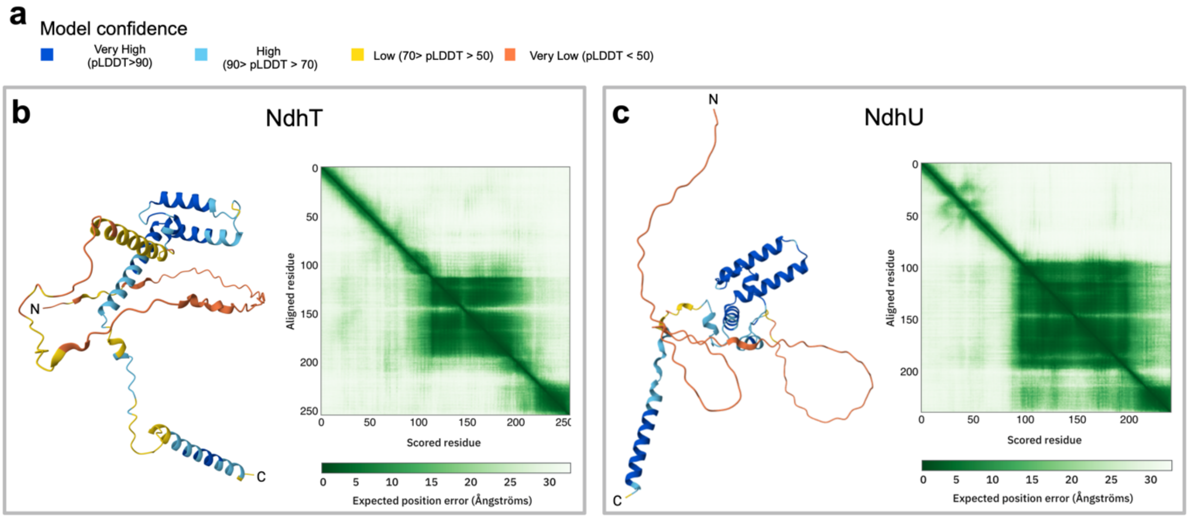

Supplementary Fig. 14: AlphaFold structure predictions and relative Predict Align Error (PAE) plots for SubB subunits. **a,** Color-key used by AlphaFold to represent the per-residue model confidence (pLDDT) for the predicted protein structure. **b-f,** In each panel, the AlphaFold predicted models (color-coded as described in **a**) are displayed on the left, with the corresponding PAE plots shown on the right. Dark green indicates high confidence (low error), while light green reflects lower confidence (higher error). The AlphaFold-predicted structures shown above served as the initial models for developing the final structure presented in this study.

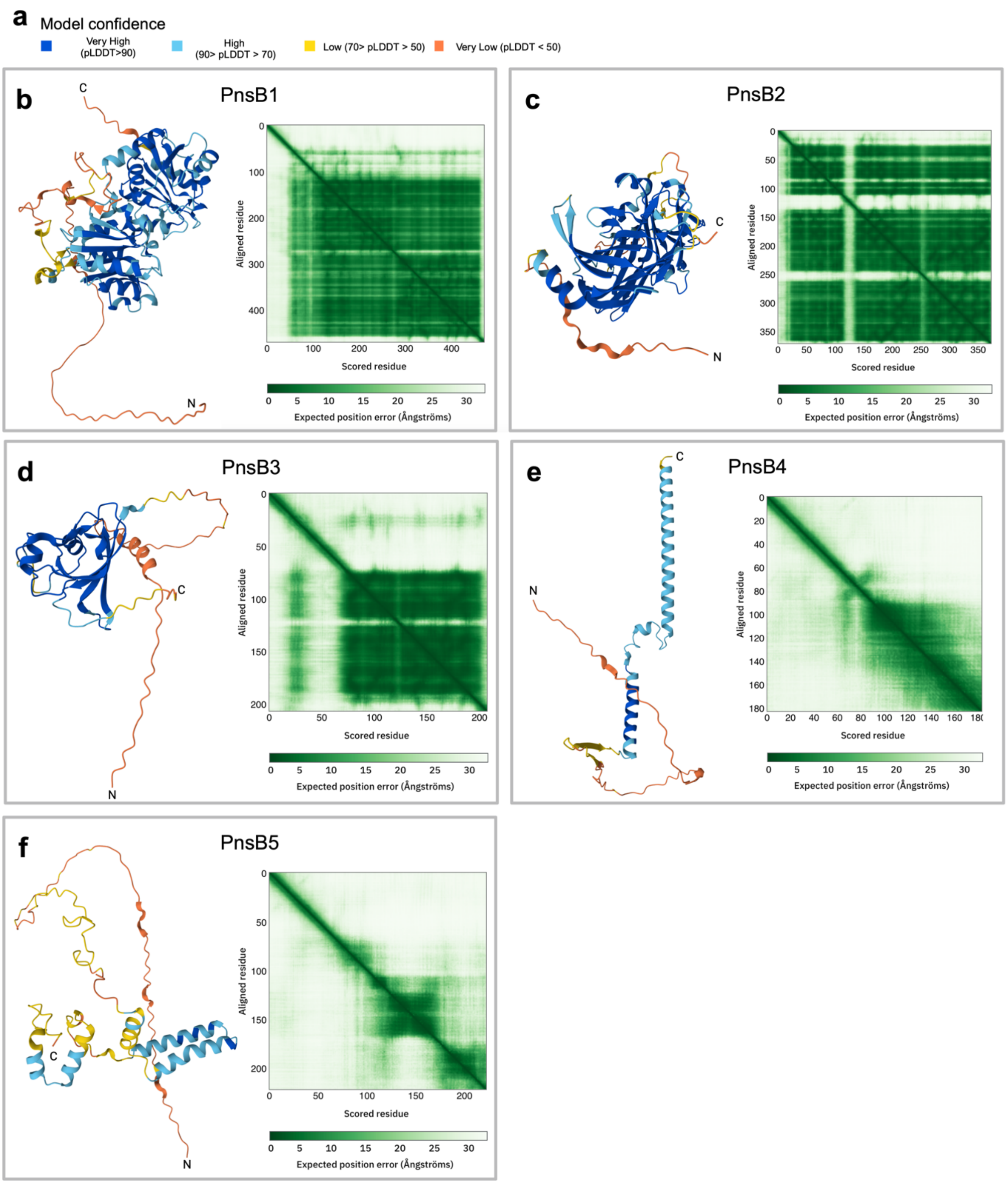

Supplementary Fig. 15: AlphaFold structure predictions and relative Predict Align Error (PAE) plots for SubL subunits. **a,** Color-key used by AlphaFold to represent the per-residue model confidence (pLDDT) for the predicted protein structure. **b-f,** In each panel, the AlphaFold predicted models (color-coded as described in **a**) are displayed on the left, with the corresponding PAE plots shown on the right. Dark green indicates high confidence (low error), while light green reflects lower confidence (higher error). The AlphaFold-predicted

structures shown above served as the initial models for developing the final structure presented in this study.

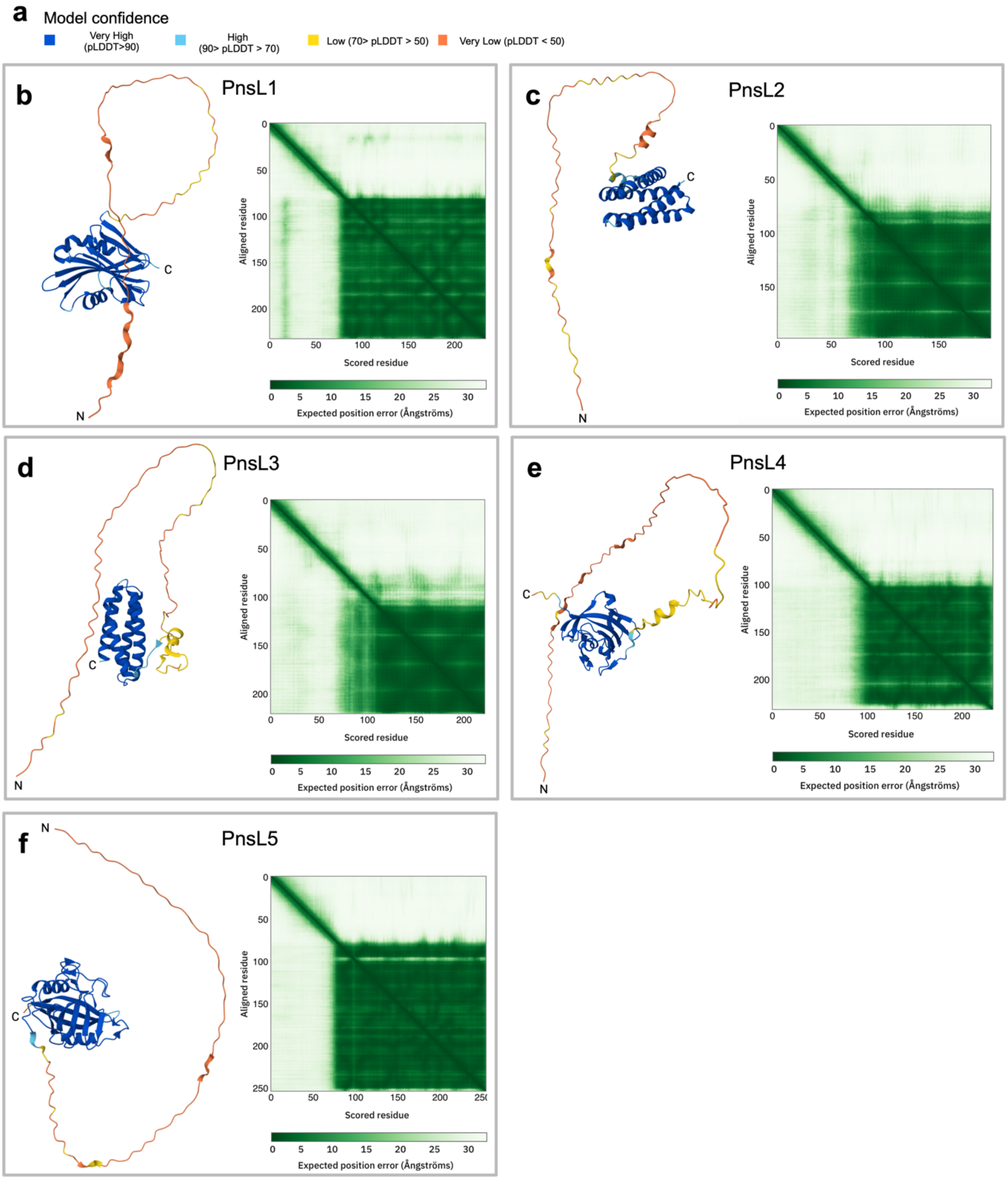

Supplementary Fig. 16: AlphaFold structure predictions and relative Predict Align Error (PAE) plots for the SubE subunits NdhT and NdhU. **a,** Color-key used by AlphaFold to represent the per-residue model confidence (pLDDT) for the predicted protein structure. In each panel, the AlphaFold predicted models for **b**, NdhT, and **c**, NdhU (color-coded as described in a) are displayed on the left, with the corresponding PAE plots shown on the right. Dark green indicates high confidence (low error), while light green reflects

lower confidence (higher error). The AlphaFold-predicted structures shown above served as the initial models for developing the final structure presented in this study.

A NDHI						Complex I				
Plants			Cyanobacteria			Plant	Fungus	Mammals		Yeast
S. oleracea	*H. vulgare*	*A. thaliana*	*T. elongatus*	*T. elongatus*	*T. elongatus*	*A. thaliana*	*C. thermophilum*	*S. domesticus*	*M. musculus*	*Y. lipolitica*
	7EU3	7WFF	6HUM	6KHJ,6LKI	6L7P,6L7O	8BEH	7ZMB	7V2C, 7V2D	6G2J,6G72	7O71,7O6Y
PG 405/A	X	X	X	DGDG	DGDG	X	X	X	X	X
PG 407/A	X	X	X	X	X	CDL	X	CDL	PC1	3PE
PG 408/A	X	X	X	X	X	X	X	X	X	X
PG 411/A	X	X	X	PG	X	X	3PE	PEE	X	3PE
PG 524/B	X	X	X	X	X	3PH	X	X	X	X
PG 525/B	X	X	X	X	PG	PTY	X	X	X	X
PG 526/B	X	X	X	X	X	PTY	LMN	X	X	X
PG 1012/D	X	X	X	PG	PG	PC7	X	CDL	X	X
PG 1013/D	PG	SQDG	X	PG	PG	X	CDL	X	X	CDL
PG 1017/F	X	X	X	X	X	3PH	3PE	X	X	X
PG 374/L	X	X	X	DGDG	DGDG	X	X	X	X	X
PG 476/L	X	X	X	SQDG	SQDG	X	PC1	PLX	PC1	PLC
PG 477/L	X	X	X	X	X	X	X	X	X	X
PG 100/4	X	X	X	X	PG	3PH	PC1	X	X	X
MGDG 410/A	X	X	X	PG	PG	PTY	3PE	PEE	3PE	3PE
MGDG 1004/D	X	X	X	X	X	PTY	X	X	X	X
MGDG 1011/D	X	X	X	X	X	X	X	X	X	X
MGDG 1016/F	X	X	X	X	X	X	3PE	X	3PE	3PE
MGDG 301/7	X	X	X	X	X	X	X	X	X	X
SQDG 522/B	X	PG	X	SQDG	SQDG	PTY	PC1	PEE	3PE	PLC
SQDG 806/D	SQDG	X	X	PG	SQDG	X	PC1	X	X	3PE
SQDG 1015/F	SQDG	PG	MGDG	X	SQDG	PG	PC1	CDL	CDL	3PE
A1H1M 1019/F	X	X	X	X	X	X	X	X	X	X
A1H1M 1020/F	X	X	X	X	DGT	X	X	CDL	3PE	X

B PSI								
Plants					Algae	Cyanobacteria		
S. oleracea	*H. vulgare*	*A. thaliana*	*P. sativum* Kalvadon	*P. sativum* Alaska	*C. reinhardtii*	*S. elongatus*	*Anabaena*	*Synechocystis SP. PCC6803*
	7EWK	7WFD	4Y28	4XK8	7ZQC	1JB0	6TCL	6HQB
PG 1062/a	X	X	X	X	X	X	X	X
PG 1063/a	PG	X	PG	PG	PG	PG	PG	PG
PG 1064/a	PG	PG	PG	PG	PG	PG	PG	PG
PG 924/b	X	X	X	X	X	X	X	X
PG 925/b	PG	PG	PG	PG	PG	X	X	X
PG 926/b	X	PG	X	X	X	X	X	X
PG 301/f	X	X	X	X	X	X	X	X
PG 942/w	PG	PG	PG	PG	PG	X	X	X
PG 931/x	PG	PG	PG	PG	PG	X	X	X
PG 926/z	X	X	X	X	X	X	X	X
MGDG 302/f	X	x	MGDG	X	X	X	X	X
MGDG 929/z	X	MGDG	MGDG	MGDG	X	X	X	X
MGDG 1025/j	MGDG	X	MGDG	X	MGDG	X	X	X
MGDG 1021/j	X	X	X	X	X	X	X	X
MGDG 940/w	X	X	X	X	MGDG	X	X	X
MGDG 914/x	X	MGDG	MGDG	MGDG	PG	X	X	X
MGDG 927/z	X	MGDG	X	X	MGDG	X	X	X
SQDG 1024/j	DGDG	MGDG	MGDG	MGDG	X	X	X	X
SQDG 941/w	X	X	X	X	X	X	X	X
SQDG 1023/w	X	X	MGDG	X	X	X	X	X
SQDG 909/y	X	PG	X	X	PG	X	X	X
DGDG 1061/a	X	X	X	X	x	X	X	X
DGDG 850/b	DGDG	DGDG	DGDG	DGDG	DGDG	MGDG	MGDG	MGDG
DGDG 921/x	MGDG	X	X	X	X	X	X	X

Supplementary table 1: Lipid positions are conserved throughout NDH and complex I and in PSI from different plants and algae. The lists show whether a lipid was observed in the organism indicated in the same position as in **(a)** NDH or **(b)** PSI. Lipid names are color-coded as follows: phosphatidylglycerol (PG), salmon; monogalactosyl diacylglycerol (MGDG), violet; sulfoquinovosyl diacylglycerol (SQDG), pink; digalactosyl diacylglycerol (DGDG), green. Zwitterionic lipids are in light yellow (i.e., phosphatidyl etanolamines (PTY, 3PE, PEE), phosphatidylcholines (PC1, PC7, PLX, PLC). The negatively charged lipids

cardiolipin (CL) and PG precursor phosphatidic acid (3PH) are red. The t-PCCαM (A1H1M), Digitonin (DGT) and LMNG (LMN) detergents are light grey.

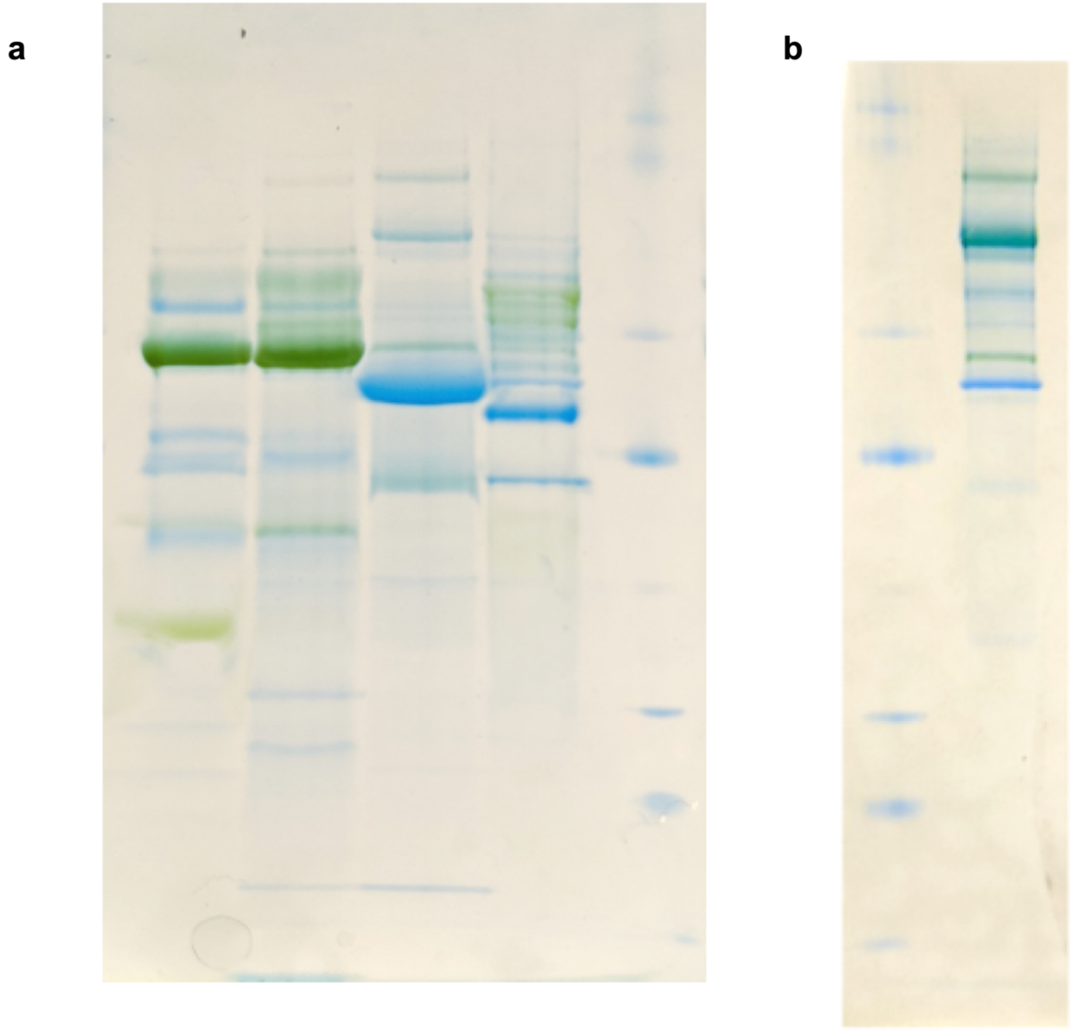

Supplementary Fig. 17: Unprocessed Gels. CN-PAGEs shown in **(a)** Supplementary Fig. 10b and **(b)** Supplementary Fig.11c.
