# Supplementary material for: Cryo-EM structure of the NDH–PSI–LHCI supercomplex from Spinacia oleracea
Source: Nat Struct Mol Biol. 2025 Jan 24;32(6):968–78. doi: 10.1038/s41594-024-01478-1 (PMC12170339; doi:10.1038/s41594-024-01478-1)
Supplement: Supplementary file 2 — Reporting Summary [file 41594_2024_1478_MOESM2_ESM.pdf]

nature portfolio

Corresponding author(s): Werner Kühlbrandt

Last updated by author(s): 28 September 2024

# Reporting Summary

Nature Portfolio wishes to improve the reproducibility of the work that we publish. This form provides structure for consistency and transparency in reporting. For further information on Nature Portfolio policies, see our Editorial Policies and the Editorial Policy Checklist.

## Statistics

For all statistical analyses, confirm that the following items are present in the figure legend, table legend, main text, or Methods section.

| n/a | Confirmed | |
|---|---|---|
| ☐ | ☒ | The exact sample size (*n*) for each experimental group/condition, given as a discrete number and unit of measurement |
| ☐ | ☒ | A statement on whether measurements were taken from distinct samples or whether the same sample was measured repeatedly |
| ☒ | ☐ | The statistical test(s) used AND whether they are one- or two-sided<br>*Only common tests should be described solely by name; describe more complex techniques in the Methods section.* |
| ☒ | ☐ | A description of all covariates tested |
| ☒ | ☐ | A description of any assumptions or corrections, such as tests of normality and adjustment for multiple comparisons |
| ☒ | ☐ | A full description of the statistical parameters including central tendency (e.g. means) or other basic estimates (e.g. regression coefficient) AND variation (e.g. standard deviation) or associated estimates of uncertainty (e.g. confidence intervals) |
| ☒ | ☐ | For null hypothesis testing, the test statistic (e.g. *F*, *t*, *r*) with confidence intervals, effect sizes, degrees of freedom and *P* value noted<br>*Give P values as exact values whenever suitable.* |
| ☒ | ☐ | For Bayesian analysis, information on the choice of priors and Markov chain Monte Carlo settings |
| ☒ | ☐ | For hierarchical and complex designs, identification of the appropriate level for tests and full reporting of outcomes |
| ☒ | ☐ | Estimates of effect sizes (e.g. Cohen's *d*, Pearson's *r*), indicating how they were calculated |

*Our web collection on statistics for biologists contains articles on many of the points above.*

## Software and code

Policy information about availability of computer code

| | |
|---|---|
| Data collection | EPU 2.8.1, EPU 2.9.0 |
| Data analysis | cryoSPARC v3.2 - cryoSPARC v4.2.1, Topaz 0.2.5a, RELION 3.0, COOT 8.0, UCSF ChimeraX 1.6, Isolde, Phenix 1.21, AlphaFold2, Molprobity 4.5.2 |

For manuscripts utilizing custom algorithms or software that are central to the research but not yet described in published literature, software must be made available to editors and reviewers. We strongly encourage code deposition in a community repository (e.g. GitHub). See the Nature Portfolio guidelines for submitting code & software for further information.

## Data

Policy information about availability of data

All manuscripts must include a data availability statement. This statement should provide the following information, where applicable:

- Accession codes, unique identifiers, or web links for publicly available datasets
- A description of any restrictions on data availability
- For clinical datasets or third party data, please ensure that the statement adheres to our policy

The maps have been deposited to EMDB: EMD-51527 (composite map, NDH-PSI-LHCI-2 supercomplex); EMD-19244 (complete map, NDH-PSI-LHCI-2 supercomplex); EMD-19241 (local refined peripheral arm of NDH); EMD-19246 (local refined membrane arm of NDH); EMD-19247 (local refined border region between NDH and PSI-LHCI-2); EMD-19248 (local refined PSI-LHCI-2). The atomic model of NDH-PSI-LHCI-2 supercomplex has been deposited with the PDB ID: 9GRX.
PDB ID: 4Y28 and PDB ID: 6KHJ were used as consensus structures for the initial construction of the here presented model.

## Research involving human participants, their data, or biological material

Policy information about studies with human participants or human data. See also policy information about sex, gender (identity/presentation), and sexual orientation and race, ethnicity and racism.

| | |
|---|---|
| Reporting on sex and gender | N/A |
| Reporting on race, ethnicity, or other socially relevant groupings | N/A |
| Population characteristics | N/A |
| Recruitment | N/A |
| Ethics oversight | N/A |

Note that full information on the approval of the study protocol must also be provided in the manuscript.

# Field-specific reporting

Please select the one below that is the best fit for your research. If you are not sure, read the appropriate sections before making your selection.

- [x] Life sciences
- [ ] Behavioural & social sciences
- [ ] Ecological, evolutionary & environmental sciences

For a reference copy of the document with all sections, see nature.com/documents/nr-reporting-summary-flat.pdf

# Life sciences study design

All studies must disclose on these points even when the disclosure is negative.

| | |
|---|---|
| Sample size | No statistical method was used to predetermine the sample size. The number of micrographs collected was determined by the observed particle distribution per image at the necessary magnification (pixel size) for reconstructing density maps at near-atomic resolution, which is essential for unbiased model building and interpretation. Following dataset classification, the remaining particles were sufficient for reconstructing a density map of the NDH-PSI-LHCI-2 supercomplex at a resolution of 3.2 Å. Additionally, local density maps were generated at resolutions of 3.3 Å, 3.2 Å, and 3.1 Å for the NDH peripheral arm, NDH membrane arm, the interface between NDH and PSI-LHCI-2, respectively. |
| Data exclusions | No data was excluded from initial data processing. Exclusion was later performed by unbiased 2D and 3D classification as part of the described data processing pipelines. Particle classes that form features-enriched 2D class averages or 3D back projections were manually selected, combined and used for further rounds of 2D/3D classification and selection. Additional details can be found in Supplementary Figures 12 and 13, as well as in the Methods section.. |
| Replication | Not applicable. Replication of entire datasets for structural biology studies is not applicable due to technical limitations. However, reconstructions of 3D maps represent an weighted average of thousands of individual particle images contained within the final dataset. |
| Randomization | We utilized the Gold standard Fourier Shell Correlation method to assess the resolution of the cryo-EM structures. This method involves splitting the dataset into two sets, odd and even, which are then refined independently. The splitting of the dataset is random. |
| Blinding | Not applicable. |

# Reporting for specific materials, systems and methods

We require information from authors about some types of materials, experimental systems and methods used in many studies. Here, indicate whether each material, system or method listed is relevant to your study. If you are not sure if a list item applies to your research, read the appropriate section before selecting a response.

## Materials & experimental systems

| n/a | Involved in the study | |
|---|---|---|
| ☒ | ☐ | Antibodies |
| ☒ | ☐ | Eukaryotic cell lines |
| ☒ | ☐ | Palaeontology and archaeology |
| ☒ | ☐ | Animals and other organisms |
| ☒ | ☐ | Clinical data |
| ☒ | ☐ | Dual use research of concern |
| ☐ | ☒ | Plants |

## Methods

| n/a | Involved in the study | |
|---|---|---|
| ☒ | ☐ | ChIP-seq |
| ☒ | ☐ | Flow cytometry |
| ☒ | ☐ | MRI-based neuroimaging |

## Plants

| | |
|---|---|
| Seed stocks | In this study, we opted not to utilize seed stock as our primary source material. Instead, we used young leaves obtained directly from market spinach (Spinacia oleracea). |
| Novel plant genotypes | N/A |
| Authentication | N/A |
